# Addressing racial and ethnic inequity in health-care, a collection

**DOI:** 10.1016/j.eclinm.2022.101372

**Published:** 2022-03-31

**Authors:** eClinicalMedicine

Health-care systems worldwide are fraught with inequalities that have a disproportionate impact on minoritised ethnic and racial groups. These inequalities may lead to reduced access to appropriate health care services, and consequently, poorer health outcomes. Furthermore, research focusing on health outcomes of racial minorities is vastly lacking. In an effort to address the need for research on racial inequality, *eClinicalMedicine* launched part 1 of an online collection entitled “*Racial Inequity in Health*” in June 2021.We have now curated part 2 of the collection, which forms part of *The Lancet's* strategy to address racial inequity in health care. The papers in this collection highlight racial and ethnic inequality across global healthcare settings, and emphasise that without targeted action, such inequalities are maintained and reinforced. The collection will therefore provide a platform for the dissemination of relevant research on racial disparities in chronic kidney disease (CKD) cardiovascular disease and maternal outcomes, amongst others. This Editorial will discuss several of the papers available in the collection.

Previous studies have shown that not only is the prevalence of CKD higher in minoritised ethnic groups, but also that it has a faster progression to end-stage renal disease in these patients compared with the majority ethnic group. Two papers in the collection explore racial and ethnic disparities in CKD in The Netherlands and the US, respectively. Huisman and colleagues showed that the estimated glomerular filtration rate (eGFR) and urinary albumin-to-creatinine ratio (ACR), which are two parameters used for the classification and prognosis evaluation of CKD, are higher among minoritised ethnic groups compared with individuals of Dutch origin. High eGFR values may precede the development of CKD. The authors suggest that current screening tools based on these parameters may therefore have important limitations in multiethnic populations and a more personalised approach is therefore required. In a similar vein, Tsai and colleagues reported that that removal of race correction from equations used to obtain the eGFR, which systematically inflate the eGFR for Black patients, would increase the percentage of Black patients diagnosed with CKD and those who are referred to specialty care and transplantation in the US.

Ethnicity has been established as an independent risk factor for cardiovascular death, alongside other risk factors such as smoking, age, blood pressure, sex, and hyperlipidaemia. Two further studies in the collection have investigated racial and ethnic disparities in cardiovascular death and vascular dysfunction, respectively. Kist and colleagues quantified the sex-specific cardiovascular death rates stratified by ethnicity and socioeconomic factors in an urban population with a universal healthcare system in The Netherlands. Large health disparities in cardiovascular age-standardised death rates were observed across ethnic and socioeconomic subgroups. The authors suggest that the identification of these high-risk subgroups as well as tailored preventative efforts may improve cardiovascular health equity within communities. In another study from The Netherlands, Hayfron-Benjamin and colleagues investigated the associations of three inflammatory biomarkers (high sensitivity C-reactive protein, fibrinogen, and D-dimer) with aortic stiffness, coronary artery disease, and peripheral artery disease in different ethnic groups. After adjusting for conventional cardiometabolic risk factors, the authors noted ethnic differences in the associations of low-grade inflammation and vascular dysfunction. This could potentially aid vascular disease prevention, diagnosis, and treatment strategies in different ethnic groups.

In addition to documenting the racial and ethnic inequity in chronic diseases, the collection also demonstrates that such disparities are seen in maternal morbidity and mortality in both high- low- and middle-income settings. Knight and colleagues described factors associated with women who died in the UK during or up to a year after the end of pregnancy, and compared the quality of care received by women from different ethnic groups to identify any structural or cultural biases or discrimination that affected their care. While the authors found no major differences in the causes of death among women from different ethnic groups, multiple areas of bias were identified in the care women received, including a lack of nuanced care, microaggressions, and differences in clinical, social and cultural factors. In Latin America, Rios-Quituizaca and colleagues showed that Ecuadorian women who self-identified as Indigenous had a lower prevalence of modern contraceptive use, antenatal care, skilled birth attendance, and institutional delivery compared with the majority ethnic group in the country. The authors stress that while gaps in care have recently narrowed, Indigenous people are still subjected to structural racism and can have limited access to health care interventions.

As evidenced above, the collection covers a breadth of medical specialties indicating that racial disparities are pervasive throughout the health-care landscape. Furthermore, the studies emphasise that minoritised ethnic groups still bear the brunt of health-care challenges. By assembling these studies in an open-access collection, we hope to empower policy makers and health-care workers to prioritise health inequalities among minoritised ethnic groups at research, clinical, and governmental levels. Commitment and systemic change are required to ensure that the health-care needs of minoritised ethnic and racial groups are met and to overcome the long-standing effects of racism.

